# Multiparametric ultrasound evaluation of a case of bilateral carotid body tumor

**DOI:** 10.1007/s40477-021-00581-z

**Published:** 2021-05-17

**Authors:** Patrizia Pacini, Giorgia Polti, Antongiulio Faggiano, Elisa Giannetta, Maria Grazia Tarsitano, Vito Cantisani

**Affiliations:** 1 Department of Radiological, Pathological and Oncological Sciences, Sapienza University of Rome, 00161 Rome, Italy; 2 Endocrinology Unit, Department of Clinical and Molecular Medicine, Sant’Andrea Hospital, Sapienza University of Rome, 00189 Rome, Italy; 3 Department of Experimental Medicine, Sapienza University of Rome, 00161 Rome, Italy

**Keywords:** Paragangliomas, Head and neck, Carotid body tumors, Ultrasound, CEUS

## Abstract

Paragangliomas are a rare form of highly vascularized tumors that originate from paraganglia Baysal (J Med Genet 39: 617–622, 2002). In the head and neck PGL arise primarily in four distinct areas: vagal, middle ear, and larynx and more frequently carotid bifurcation. Imaging evaluations include sonography, color Doppler, US-elastosonography and contrast-enhanced ultrasound (CEUS). Additionally, Computed Tomography, Magnetic Resonance Imaging (MRI) as well as digital subtraction angiography can be performed Stoeckli et al. (Laryngoscope 112: 143–146, 2002). We present herein a case of a rare bilateral carotid body tumor assessed with multiparametric ultrasound evaluation, including CEUS and US-elastography.

## Introduction

**Fig. 1:**
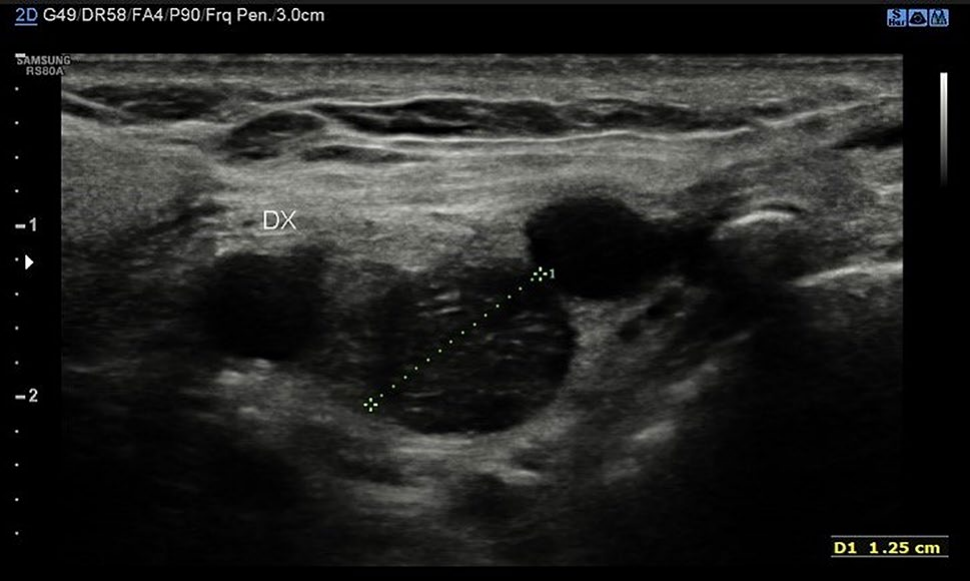
Rounded mass with mixed echogenicity and defined margins, localized in the carotid bifurcation

**Fig. 2:**
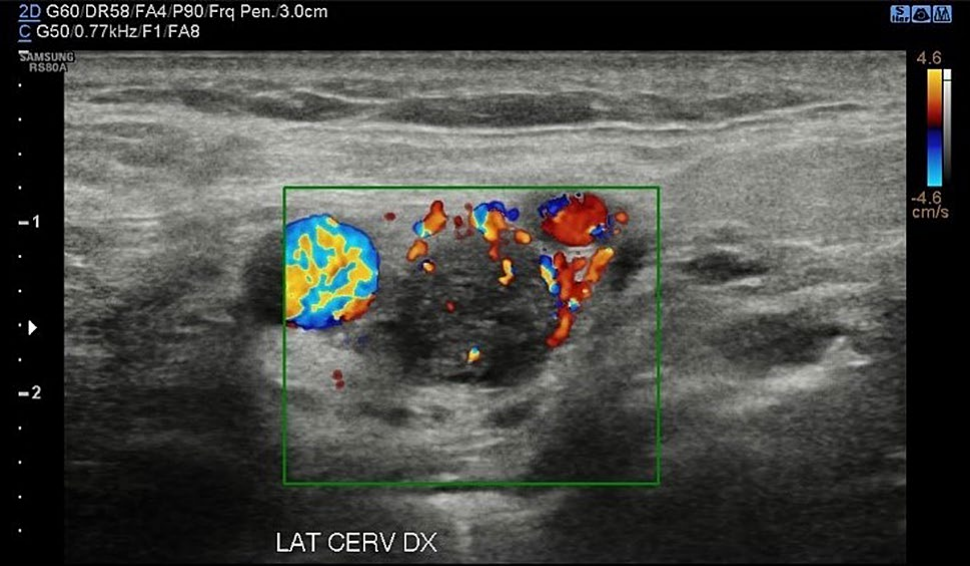
A predominantly peripheral vascularization with some centripetal vessels at Color-doppler

**Fig. 3:**
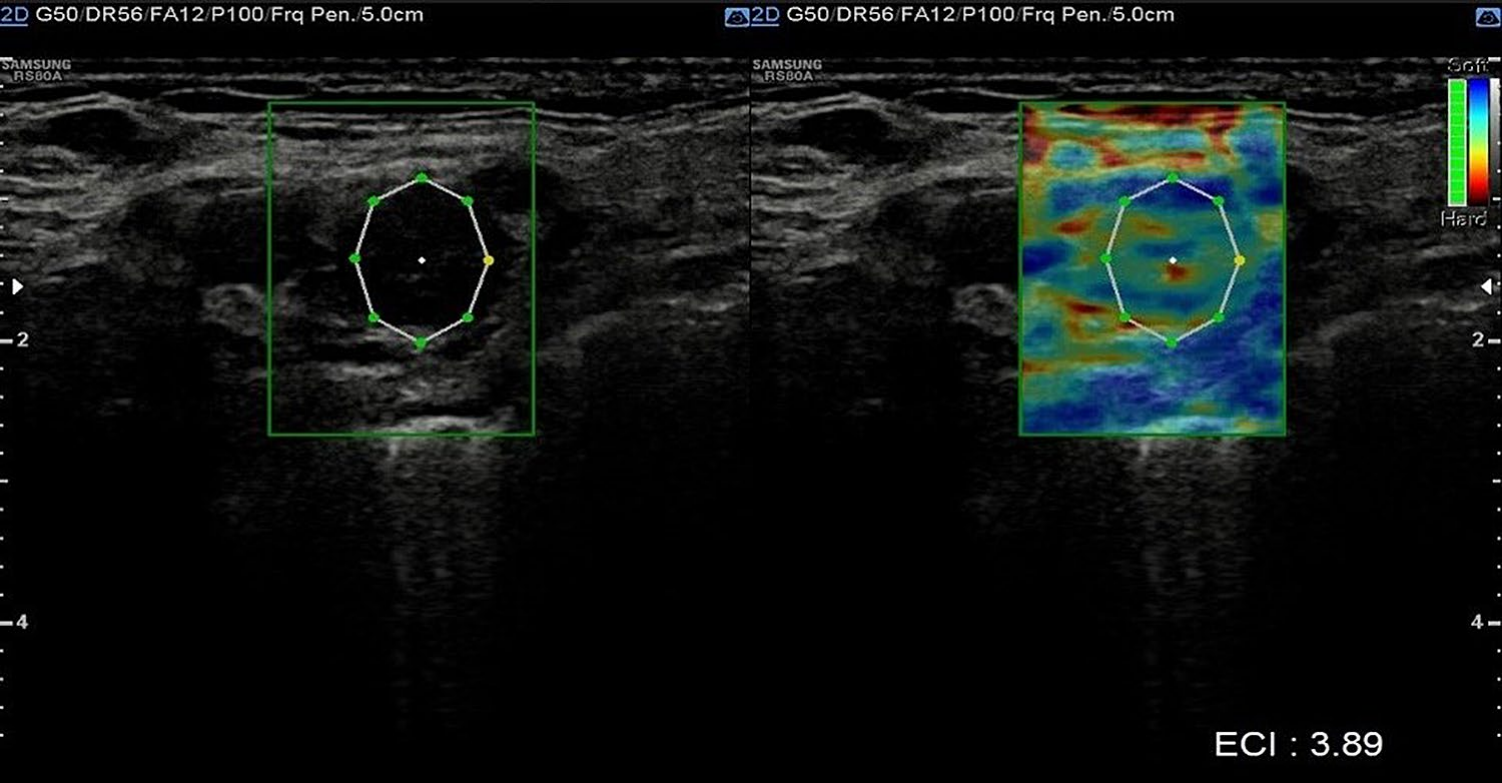
At the US-elastography, the lesion appears stiff

**Fig. 4:**
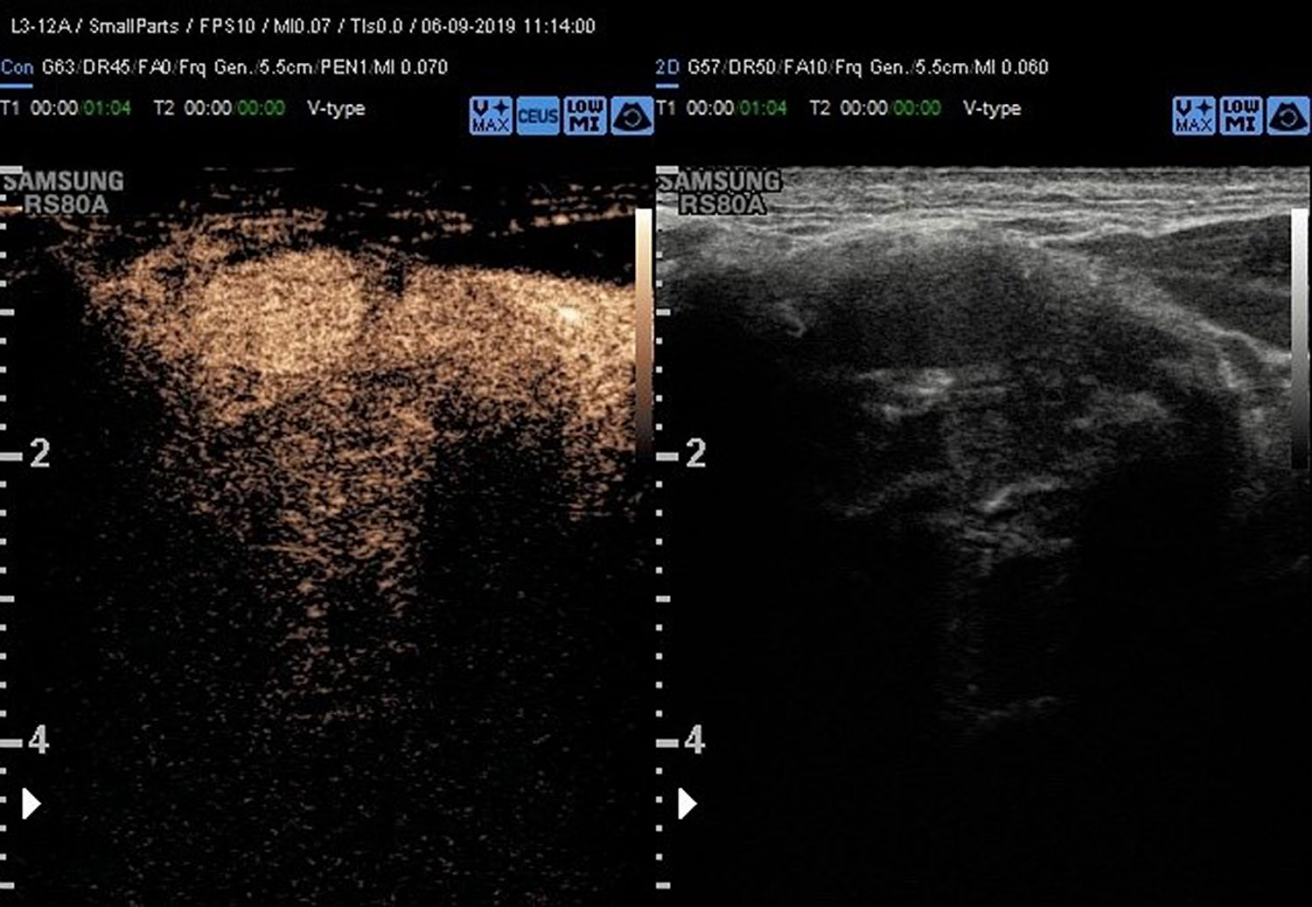
CEUS examination revealed a homogeneous enhancement of the lesion with slow and progressive wash out as showed by Intensity-Time Curve in Fig. 5

**Fig. 5:**
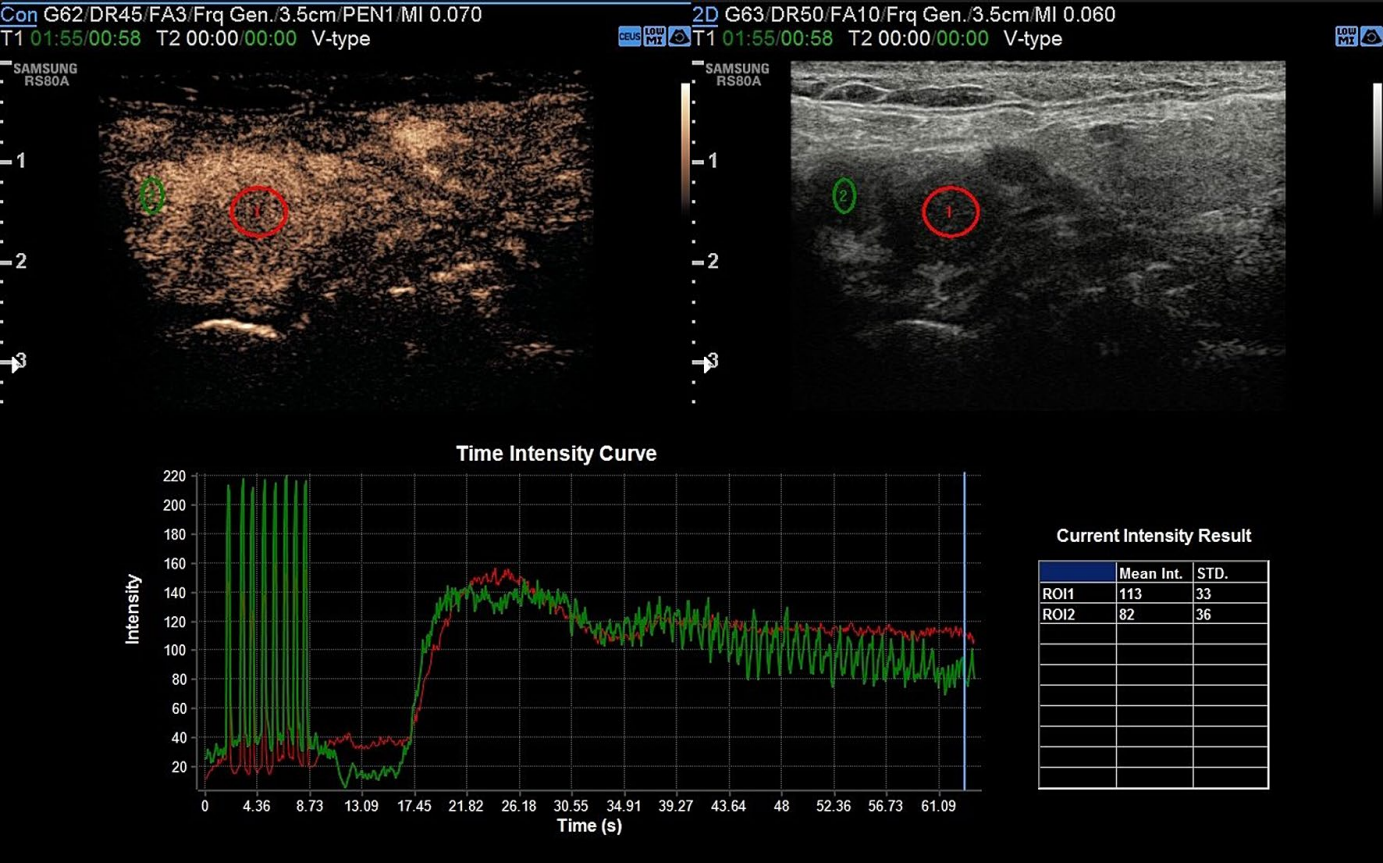
Quantitative measurement using the Intensity-Time Curve

Head and neck paragangliomas (HNPGL) are a group of slow-growing hypervascular tumors associated with the paraganglion system [3], occurring at a rate of 0.03% of all tumors and 0.6% of all head and neck tumors. Radical surgery had been the traditional approach, but improvements in diagnostic imaging have opened new possibilities of nonsurgical therapy such as radiation therapy techniques in many patients or even medical therapy in selected cases [4]. HNPGLs most commonly represent a hereditary familial condition [5, 6]. The predominant pathway involved in HNPGL tumorigenesis is the succinate dehydrogenase (SDH) enzyme, which is a multiprotein complex composed of SDHA, SDHB, SDHC, and SDHD proteins in addition to SDHAF2. PGLs arise from the paraganglia of the parasympathetic system in the HN and are rarely functional with an estimated prevalence of 1–9 / 1 000 000 and the management is still not universally standardized. From a biological point of view, HNPGL are neuroendocrine tumors arising from paraganglia, slowly growing for the most but potentially locally invasive. After identification of the case index, a genetic screening is performed in all first-degree relatives. All carriers for SDH mutations need to undergo a clinical and radiological work-up. An, accurate and fast diagnosis is crucial for a prompt and reliable treatment of PGLs. The gold standard is the post-surgical histological exam, but several imaging modalities have been proposed for the work-up of these patients, and among them, B-mode and color Doppler- sonography, uselastography and contrast-enhanced ultrasound (CEUS). Additionally, Computed Tomography (CT), Magnetic Resonance Imaging, 123I-Metaiodobenzylguanidine (MIBG) scintigraphy, PET-TC as well as digital subtraction angiography can be performed [2]. To the best of our knowledge, we present herein the second case of bilateral carotid body tumor studied with CEUS. Case presentation A 66 year-old Caucasian male presented to our observation with a painless, slowly growing swelling in the left lateral region, incidentally identified while shaving. The overlying skin was normal and there were no clinical signs referring to facial nerve injury. Patient’s anamnesis did not reveal other oncological history or a similar case in his family. He came to our Radiology Department attention to perform a B-mode US evaluation as first-level assessment which showed: at Bmode a rounded lesion, with defined margins, with mixed echogenicity, approximately of 4 cm, localized in the carotid bifurcation (Fig. 1); color Doppler evaluation highlighted a predominantly peripheral vascularization with some centripetal vessels inside the lesion (Fig. 2). The patient subsequently was assessed by means of USE and the lesion appeared of hard consistency (Fig. 3); then at CEUS after administration 2 ml of intravenous contrast medium (Sonovue, Bracco, Milan, Italy), followed by 10 ml of isotonic saline solution the lesion presented homogeneous enhancement with enhancement similar to that of the internal carotid artery (Fig. 4), also confirmed at quantitative measurement using the Intensity-Time Curve (Fig. 5). Contralaterally, another smaller lesion with similar findings was identified. The final diagnosis of Glomus Carotid Paraganglioma was confirmed at post-surgical histology.

## Discussion and conclusions

Familial paraganglioma syndrome type 1 (FPGL1) is due to mutation in SDHD gene and it is characterized by the development of PGLs, in the most of cases localized in head and neck region. PGLs normally present after the age of 30 years [7]. Clinical presentation of the CBT is an asymptomatic slowly growing mass in neck that usually identified by clinical examination or found incidentally on imaging studies; rarely the lesion can produce symptoms due to pressure and local invasion of the surrounding tissue. Clinical history, examination, and radiological diagnosis are the keystones to diagnosis and management. Multiparametric ultrasound with CEUS and USE, CT, MRI, and angiography play important roles in the clinical diagnosis of CBT. Color Doppler ultrasound, a simple and non-invasive examination, has relatively high specificity and sensitivity for CBT. Color-flow carotid duplex is the ideal screening test for CBTs, and these tumors are characteristically a well-defined hypoechoic mass that displaces the carotid bifurcation. With color Doppler imaging, a hypervascularity with low-resistance flow pattern is evident.

The most recent EFSUMB guideline describes USE [8] as a technique that requires mechanical or acoustic pressure on the organ of interest to measure the shear deformations of the internal tissue resulting from this pressure. About the CBT there is not yet a clear and defined pattern the tumor it can be both soft and hard. In our case report the lesion appears of hard consistency.

Historically, angiography was the “gold standard” diagnostic procedure for CBTs to confirm the diagnosis and provide accurate delineation of the vascular supply. However, with improvements in high-resolution imaging, cross-sectional imaging (such as CT angiography or MR angiography) is the preferred modality for surgical planning of tumor resection because it best defines the relationship of the tumor with the artery bifurcation and the likely location of the cranial nerves. MRI does not use ionizing radiation, and the accuracy of MRI is higher in comparison to that of CT in many cases.

Although, CEUS has a recognized role for different organs [9–11]. There is still limited evidence on the use of CEUS in the head and neck paraganglioma [12, 13] and this is a second case of PGL published with CEUS and the first bilateral with multiparametric US. Our case has subtle differences from the further case of neck paraganglioma already described [12], presenting a mixed echogenicity at B-mode exam (versus hypoechogenicity), a predominantly peripheral vascularization with some centripetal vessels at the color Doppler evaluation (versus no vascularization in the middle) and a homogeneous enhancement similar to that of the internal carotid artery after administration of mdc.

Indeed, CEUS appears a useful and safe tool for identifying CBTs and evaluating intratumoral microperfusion at high spatial and temporal resolutions in real-time, although it should be taken in account the main limitation that is the lack of panoramicity. In the daily clinical routine CEUS can be used to easily monitor therapy success and efficiency after embolization and seems to be a viable option with superior benefits compared to standard Color-Doppler for follow-up of these patients. Additionally, CEUS can be performed on almost every patient because of the limited contraindications of the used contrast agent. Patients with reduced renal function can particularly benefit from this method [11]. In the recent guidelines published in Ultraschall on the CEUS use for non-hepatic applications, it has been reported that nneuroendocrine tumors typically present as hyper-enhancing lesions in the arterial phase of CEUS examinations, owing to their significant arterialization and that based on the ENETs Consensus Guidelines, CEUS has been suggested as an imaging modality for the diagnosis of neuroendocrine neoplasms[10]

In conclusion CEUS may be implemented in the future diagnostic work-up and follow-up of CBT patients in addition to conventional ultrasound, CT, MRI and digital substraction angiography (DSA). Therefore, further multi-center studies are encouraged to confirm the clinical use of these techniques on CBTs, as limited evidence is still available.
